# Endoproteolysis of cellular prion protein by plasmin hinders propagation of prions

**DOI:** 10.3389/fnmol.2022.990136

**Published:** 2022-09-02

**Authors:** Charles E. Mays, Trang H. T. Trinh, Glenn Telling, Hae-Eun Kang, Chongsuk Ryou

**Affiliations:** ^1^Department of Microbiology, Immunology, and Molecular Genetics, University of Kentucky College of Medicine, Lexington, KY, United States; ^2^Department of Pharmacy, College of Pharmacy, Hanyang University, Ansan, South Korea; ^3^Institute of Pharmaceutical Science and Technology, Hanyang University, Ansan, South Korea; ^4^Sanders-Brown Center on Aging, University of Kentucky College of Medicine, Lexington, KY, United States; ^5^Department of Microbiology, Immunology, and Pathology, Colorado State University, Fort Collins, CO, United States; ^6^Reference Laboratory for Chronic Wasting Disease (CWD), Foreign Animal Disease Division, Animal and Plant Quarantine Agency, Gimcheon, South Korea

**Keywords:** plasmin, prion, endoproteolysis, α-cleavage, PrP*^Sc^* propagation

## Abstract

Many questions surround the underlying mechanism for the differential metabolic processing observed for the prion protein (PrP) in healthy and prion-infected mammals. Foremost, the physiological α-cleavage of PrP interrupts a region critical for both toxicity and conversion of cellular PrP (PrP*^C^*) into its misfolded pathogenic isoform (PrP*^Sc^*) by generating a glycosylphosphatidylinositol (GPI)-anchored C1 fragment. During prion diseases, alternative β-cleavage of PrP becomes prominent, producing a GPI-anchored C2 fragment with this particular region intact. It remains unexplored whether physical up-regulation of α-cleavage can inhibit disease progression. Furthermore, several pieces of evidence indicate that a disintegrin and metalloproteinase (ADAM) 10 and ADAM17 play a much smaller role in the α-cleavage of PrP*^C^* than originally believed, thus presenting the need to identify the primary protease(s) responsible. For this purpose, we characterized the ability of plasmin to perform PrP α-cleavage. Then, we conducted functional assays using protein misfolding cyclic amplification (PMCA) and prion-infected cell lines to clarify the role of plasmin-mediated α-cleavage during prion propagation. Here, we demonstrated an inhibitory role of plasmin for PrP*^Sc^* formation through PrP α-cleavage that increased C1 fragments resulting in reduced prion conversion compared with non-treated PMCA and cell cultures. The reduction of prion infectious titer in the bioassay of plasmin-treated PMCA material also supported the inhibitory role of plasmin on PrP*^Sc^* replication. Our results suggest that plasmin-mediated endoproteolytic cleavage of PrP may be an important event to prevent prion propagation.

## Introduction

Prion diseases are a category of ultimately fatal neurodegenerative disorders that include Creutzfeldt–Jakob disease (CJD) in humans, bovine spongiform encephalopathy (BSE) in cattle, scrapie in sheep, and chronic wasting disease (CWD) in deer. The infectious agent responsible, termed a prion, is a completely proteinaceous particle that lacks a nucleic acid component (Prusiner, 1998). Prion propagation is characterized by a major conformational alteration for the host-encoded cellular prion protein (PrP*^C^*) to a misfolded isoform called scrapie prion protein (PrP*^Sc^*), where transition of α-helices of PrP*^C^* to β-sheets occurs (Pan et al., 1993). A hallmark of PrP*^Sc^* is its relative resistance to protease digestion *in vitro*. Proteinase K (PK) treatment of PrP*^Sc^* results in the partially resistant molecule, referred to as PrP27-30, consisting predominantly of amino acid residues 90-231 (Chen et al., 1995). In contrast to PrP*^Sc^*, PrP*^C^* is sensitive to proteolytic digestion by PK.

In addition to these biochemical differences, PrP*^C^* and PrP*^Sc^* are subject to diverse intracellular proteolytic processing events. The α-cleavage site that produces the C1 fragment (C1) was mapped to a variable site N-terminal to the hydrophobic domain within Lys110, His111, or Met112 [human (h) PrP*^C^* sequence nomenclature], while the generation of the C2 fragment (C2) by β-cleavage was estimated to occur C-terminal to the octapeptide repeat region in the vicinity of residue 90 (Chen et al., 1995; Mangé et al., 2004; Oliveira-Martins et al., 2010). A central event in PrP conformational conversion is likely metabolic processing because the dominant C-terminal PrP endoproteolytic product switches from the normal C1 to the alternative C2 as prion disease progresses (Harris et al., 1993; Chen et al., 1995; Hachiya et al., 2007). The C2 is likely to correspond to the PrP27-30 generated by PK digestion. Therefore, disruption of the neurotoxic and amyloidogenic PrP(106-126) domain by α-cleavage may prevent the accumulation of the C2 and inhibit PrP*^Sc^* propagation (Chen et al., 1995; Jimenez-Huete et al., 1998). Besides these well-described internal cleavage events, γ-cleavage of PrP*^C^* results in a soluble N-terminal fragment (N3) of 20 kDa and a small GPI-anchored C3 fragment (C3) of 5 kDa, cleaved in a region between amino acid residues 170 and 200, near N-terminal of the first N-glycosylation site (Haigh and Collins, 2016). It appears to be associated with pathophysiological conditions, as increased C3 fragments are found in CJD brain samples (Kojima et al., 2014; Lewis et al., 2016).

However, the exact mechanism for PrP processing remains unclear. To date, a handful of proteases have been reported as having the ability to cleave at the α-site: ADAM8 (Liang et al., 2012), ADAM10 (Vincent et al., 2001; Cisse et al., 2005), ADAM17 (Vincent et al., 2001; Laffont-Proust et al., 2005), calpain (Hachiya et al., 2011), and plasmin (Kornblatt et al., 2003; Praus et al., 2003; Xanthopoulos et al., 2005). Unfortunately, ADAM family proteases showed higher complexity with different cleavages dependent on divalent ions (McDonald et al., 2014); ADAM8 appears to be specifically active in extraneural tissues such as muscle (Liang et al., 2012); ADAM10 plays a larger role in shedding, presumably generating N3 (Endres et al., 2009; Taylor et al., 2009; Altmeppen et al., 2011); ADAM17 contributes less frequently than ADAM10 through a pathway regulated by protein kinase C (Vincent et al., 2000; Cisse et al., 2005). The α-cleavage of PrP by calpain is dubious because of its shared ability to perform β-cleavage (Yadavalli et al., 2004).

Plasminogen is an enzymatically inactive, kringle domain-containing zymogen expressed in the liver and to a lesser extent in the brain (Basham and Seeds, 2001; Zhang et al., 2002; Kwon and Waisman, 2003; Ledesma et al., 2003; Kim et al., 2009). Plasminogen gives rise to several biologically active fragments upon proteolytic cleavage including the serine protease plasmin (Castellino and Ploplis, 2003) that is capable of processing recombinant (r) PrP and purified PrP*^C^ in vitro* at the α-cleavage site (Kornblatt et al., 2003; Praus et al., 2003). However, the details to confirm plasmin-mediated α-cleavage of PrP remain to be further investigated because the level of C1 under plasmin-deficient condition in plasminogen null mice was found to be comparable to the C1 level in wild type mice (Barnewitz et al., 2006). Separately, a line of studies suggested that PrP*^C^* plays a role to stimulate activation to plasmin from plasminogen (Ellis et al., 2002; Praus et al., 2003; Epple et al., 2004a,b; Borumand and Ellis, 2022).

Numerous studies, including a report from one of us (Ryou et al., 2003), have described plasminogen as a PrP ligand and its interaction with PrP is mediated through the lysine-binding motifs of the plasminogen kringle domains (Fischer et al., 2000; Maissen et al., 2001; Ellis et al., 2002; Shaked et al., 2002; Kornblatt et al., 2003, 2004; Praus et al., 2003; Epple et al., 2004a,b; Cuccioloni et al., 2005; Hatcher et al., 2009; Bougard et al., 2016). Previously, we expanded on this discovery by demonstrating the ability of plasminogen to stimulate PrP*^Sc^* formation in cultured cells chronically infected with prions as well as in a cell-free system called protein misfolding cyclic amplification (PMCA) (Mays and Ryou, 2010). Furthermore, disruption of the PrP-plasminogen interaction with L-lysine and its polymers successfully inhibited PrP*^Sc^* formation in PMCA, cell culture, and mouse models of prion disease (Ryou et al., 2011). Collectively, these studies validated plasminogen as a cellular protein auxiliary factor proven to stimulate PrP*^Sc^* propagation (Mays and Ryou, 2011). However, the use of plasminogen-deficient mouse models has generated incongruent data on the role of plasminogen during disease progression. Prion challenge to plasminogen null mice results in no major change of mean incubation period of disease (Salmona et al., 2005; Xanthopoulos et al., 2005), concluding that plasminogen is not functional to control prion propagation *in vivo*. Nevertheless, one must understand that the ablation of plasminogen gene drives the deficiency of both plasminogen and plasmin and reason whether ablation of two factors (plasminogen and plasmin) with potentially opposing roles would offset each other.

We offer a “yin-yang” hypothesis for the plasmin(ogen) system, where plasminogen accelerating PrP*^Sc^* replication is counteracted by enzymatically active plasmin, cleaving PrP*^C^* at the α-site to generate the C1 fragment. Thus, we assessed the ability of plasmin to generate the endoproteolytic products for PrP and whether active up-regulation of α-cleavage by plasmin can inhibit PrP*^Sc^* propagation. Plasmin-mediated endoproteolysis of PrP was investigated using recombinant prion protein (rPrP), PrP*^C^* of prion-infected and –uninfected cultured cells, and PMCA products. To specifically address plasmin-mediated inhibition of prion propagation, we investigated PrP*^Sc^* formation in biological conditions by monitoring the accumulation of PrP*^Sc^* in prion-infected cell lines cultured with supplemented plasmin. Furthermore, we determined whether inhibition of prion propagation by plasmin can be recapitulated using the controlled conditions of PMCA, which avoids problems associated with plasminogen knockout animal models. Finally, we determined whether plasmin-facilitated inhibition of prion propagation affects prion infectivity using bioassay.

## Materials and methods

### *In vitro* plasmin cleavage for recombinant prion protein

His-tagged recombinant human (rh) PrP(23-231) was expressed and purified as previously described (Kim et al., 2014). Briefly, *E. coli* BL21(DE3)/RIL + cells (Invitrogen, Carlsbad, CA, United States) were transformed with the recombinant expression plasmid in which the encoding DNA fragment for hPrP(23-231) was cloned in pET100/D-TOPO (Invitrogen). The protein expression was induced at an OD_600_ ∼ 0.6, and grown at 37°C for 2 h. rhPrP(23-231) was purified from the lysates of harvested cells via a nickel chelate affinity resin. Three μg recombinant protein was digested with 0.05, 0.1, 0.2, or 0.5 μM human plasmin (hPln, HCPM-0140, Haematologic Technologies, Inc, Essex Junction, VT, United States) for 4 h at 37°C in a volume brought to 30 μl with Dulbecco’s Modified Eagle’s Medium (DMEM, Invitrogen) with 10% fetal bovine serum (FBS, Invitrogen). Western blot analysis or silver staining was used to analyze 1 μg aliquot samples that were loaded and run in 12–14% Tris-glycine SDS-PAGE gels. rhPrP(23-231) (0.05 μg), not mixed with FBS and hPln, was also examined. Gels for silver staining were processed as directed by the ProteoSilver™ Silver Stain Kit (Sigma-Aldrich, St. Louis, MO, United States), while gels for western blot analysis were subsequently transferred to a PVDF membrane (Immobilon-FL, Millipore, Billerica, MA, United States) as described in detail below.

### Cell culture

The Neuro2a neuroblastoma (N2a) cells (ATCC CCL-131) and scrapie-infected N2a (ScN2a) cells (Butler et al., 1988) were grown in DMEM-high glucose containing 10% inactivated FBS, 1% glutamax, and 1% streptomycin/penicillin (Invitrogen) in the presence of 5% CO_2_ and humidity at 37°C.

### Plasmin cleavage for cellular prion protein of cultured cells

For the study of PrP*^C^* cleavage in response to plasmin, cells were passed into 6-well culture plates (Corning, Lowell, MA. United States) and maintained in serum-free DMEM with 1X N-2 supplement (Invitrogen) for 18 h in the presence of 0 or 0.1 μM hPln. Cell lysate prepared with lysis buffer (20 mM Tris, pH 8.0, 150 mM NaCl, 0.5% Non-idet-P 40 and 0.5% sodium deoxycholate) was used for digestion with or without 20 μg/ml PK for 1 h at 37°C. To investigate the fragmentation of PrP*^C^* under the deglycosylated condition, PK-digested or –undigested cell lysate was further incubated with Peptide:N-Glycosidases F (PNGase F, NEB, Ipswich, MA, United States) according to manufacturer’s instruction. Briefly, the samples were first incubated in denaturing buffer (0.5% SDS, 40 mM DL-dithiothreitol) for 10 min at 100°C and further incubated with ∼1000 U of PNGase F in 50 mM sodium phosphate buffer (pH 7.5) supplemented with 1% Non-idet-P 40 for 3.5 h at 37°C. The samples were analyzed by SDS-PAGE followed by Western blotting using anti-PrP antibodies.

### Scrapie prion protein assay in cultured cells incubated with plasmin

For the study of PrP*^Sc^* propagation in response to plasmin, cells were passed into 6-well culture plates (Corning) at an estimated 5% confluence and supplemented with the specified concentration (0 – 0.5 μM) of each treatment for 6 d. After 3 d, media was replaced with fresh media and reagents. On day 6, lysates were prepared from cell cultures with lysis buffer (20 mM Tris, pH 8.0, 150 mM NaCl, 0.5% Non-idet-P 40 and 0.5% sodium deoxycholate). *De novo* formation of PrP*^Sc^* in ScN2a cells was monitored by transiently transfecting with plasmids harboring the 3F4 epitope-tagged PrP (PrP-3F4) (Scott et al., 1992). ScN2a cells maintained in Minimal Essential Media (MEM, Invitrogen) containing 10% inactivated FBS, 1% penicillin-streptomycin, and 1% glutamax were cultured in a 12-well plate (Corning) at approximately 70% confluence. Cells were transfected with 5 μg of plasmid DNA expressing PrP-3F4 or an empty plasmid vector using transfection reagent DOTAP (Roche, Basel, Switzerland). Then, at 1 d post-transfection, they were washed twice with PBS and cultured in MEM with 2% FBS for 3 d in the presence of 0 – 0.3 μM hPln. Cell lysate was prepared as described above. Analysis of PK-digested and –undigested samples was performed by the same protocol described above.

### Protein misfolding cyclic amplification

All brain materials for PMCA were obtained from 5- to 9-week-old CD-1 mice (Harlan Laboratories, Indianapolis, IN, United States) the following perfusion with phosphate-buffered saline (PBS) containing 5 mM EDTA. PrP*^C^* substrate was made by homogenizing healthy brain material 10% (w/v) in PMCA buffer [PBS, pH 7.2; 150 mM NaCl; 1% Triton X-100; 4 mM EDTA; and Complete Mini (Roche) protease inhibitors]. PrP*^Sc^* seeds were generated by homogenizing RML prion-infected brain material 10% (w/v) in PBS. To remove debris, the homogenate was centrifuged at 2000 × *g* for 5 min at 4°C. The supernatant was saved at −80°C for PMCA, while the pellet was discarded.

Our method for automated PMCA was conducted as previously described (Mays and Ryou, 2010). Briefly, PrP*^Sc^* seeds were diluted 500- to 16000-fold in PrP*^C^* substrate in a 96-well PCR plate (TempPlate III, United States Scientific, Ocala, FL, United States). Purified hPln (0–1.0 μM) or mouse plasmin (0.5 μM, mPln, MCPM-5140, Haematologic Technologies, Inc) was added in the mixture. If necessary, aprotinin (0 – 8.0 μM, A1153, Sigma-Aldrich), phenylmethylsulfonyl fluoride (PMSF, 1 mM, P7626, Sigma-Aldrich), Pefabloc SC (1 mM, Roche), or E-64 (1 mM, E3132, Sigma-Aldrich) was added as described in the text. Pre-PMCA aliquots were taken from each sample to be saved at −20°C, while the remaining mixture underwent the amplification procedure. The plates were immersed in water maintained at 37°C on the microplate horn of a microsonicator (Misonix Model 3000, Farmingdale, New York, United States) programmed for 48 h with a 40 s pulse of sonication every 30 min at a potency of 7.

Pre- and post-PMCA samples were PK-digested with 20 μg/ml PK for 1 h at 37°C and analyzed by western blotting using anti-PrP antibodies. PK-undigested, plain post-PMCA samples were further analyzed before and after PNGase F digestion, which was performed as described above.

### Prion bioassay

The assay was performed as described elsewhere (Ryou et al., 2011). Age-matched groups of 5 to 6-week-old female CD-1 mice (Harlan Laboratories, Indianapolis, IN, United States) were intracerebrally inoculated with PMCA material diluted 1:10 in PBS. During this procedure, isoflurane was utilized to anesthetize animals to minimize pain or discomfort. While the animal was unconscious, intracerebral inoculation was performed by injection at a depth of 2-3 mm with 30 μl of inoculum using a 26-gauge needle inserted in the middle of the left parietal lobe beside the midline. Incubation time was measured by determining the point at which multiple characteristic disease signs (including slowed movement, hunched posture, increased tone of the tail, loss of balance, and roughened coat) had progressed to the terminal stage, and the brains of the prion-infected mice were collected at this time. This protocol was approved by the Institutional Animal Care and Use Committee, University of Kentucky. Ten% (w/v) homogenate prepared from the collected brains was digested in PBS including 2% Sarkosyl with 20 μg/ml PK at 37°C for 1 h and analyzed by western blotting.

### Western blotting

Immunoblotting for recombinant protein, cell lysates, PMCA samples, brain material, and their PK- or PNGase F-digested samples was performed by separation on a 12–14% Tris-glycine SDS-PAGE gel followed by western blotting as previously described (Mays and Ryou, 2010). Following gel electrophoresis and electrotransfer of proteins in the gel to PVDF membrane, blocking for 1 h with 5% skim milk was carried out. The membranes were immediately incubated with anti-β-actin antibody (ACTN05; Neomarker, Fremont, CA, United States), anti-PrP antibodies 3F4 (Signet Laboratory, Boston, MA, United States), D13, or D18 (gifted by Dr. Stanley Prusiner, University of California, San Francisco, CA, United States). ECL Plus kit (GE Healthcare, Amersham Biosciences, Piscataway, NJ, United States) was used for chemiluminescence detection. The blots were visualized by developing on film or scanning with the Fuji Film FLA 5000 image reader (Fuji Film, Edison, NJ, United States).

### Histopathologic analysis

Histology and immunohistochemistry were performed according to the methods described elsewhere (Nazor et al., 2007). Briefly, brains were dissected rapidly after sacrifice of the animal and immersion fixed in 10% buffered formalin. Tissues were embedded in paraffin and 10 μm-thick coronal microtome sections were mounted onto positively charged glass slides and stained with hematoxylin and eosin for evaluation of spongiform degeneration. Following inactivation of endogenous peroxidases by incubation in 3% H_2_O_2_ in methanol, peroxidase immunohistochemistry was used to evaluate the extent of reactive astrocytic gliosis using to anti-glial fibrillary acidic protein (GFAP) antibodies (Abcam, Cambridge, United Kingdom). Detection was with Vectastain ABC reagents (Vector Laboratories, Newark, CA, United States) and slides were developed with diaminobenzidine.

### Statistical analysis

The Kaplan-Meier survival curve was calculated by SigmaPlot 11.0. Statistical analysis of difference in incubation time between experimental groups was performed using the log-rank test. *P* < 0.05 was considered statistically significant.

## Results

### Plasmin internally cleaves recombinant prion protein

**FIGURE 1:**
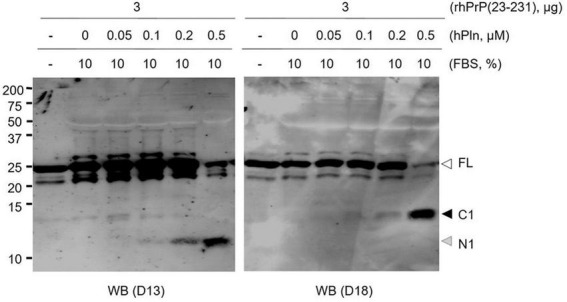
Plasmin cleaves rPrP *in vitro*. rhPrP(23-231) was treated with increasing concentrations of hPln in DMEM with 10% FBS. Western blot analysis was utilized to visualize plasmin-generated C-terminal (D18) and N-terminal (D13) fragments for rPrP. white arrow; full-length (FL) rPrP; black arrow, C1 fragment; gray arrow, N1 fragment.

Several studies reported that plasmin is able to cleave the PrP at lysine residue 110 generating an N-terminally truncated molecule that has previously been described as a major product of PrP*^C^* metabolism (Praus et al., 2003). To address the specific proteolytic activity of plasmin, we conducted *in vitro* plasmin cleavage of rPrP in the serum conditioned buffer. rhPrP(23-231) was subjected to increasing hPln concentrations ranging from 0 to 0.5 μM. In agreement with other studies (Kornblatt et al., 2003; Praus et al., 2003; Xanthopoulos et al., 2005), antibody mapping of plasmin-digested full-length rhPrP(23-231) suggested the α-cleavage generation of an 11 kDa N-terminal fragment [N1, likely PrP amino acid residues (aa) 23-110] and a larger 13 kDa C-terminal fragment (C1, likely PrP aa 111-231). D13 antibody recognizing PrP aa 94-105 and D18 antibody recognizing PrP aa 133-157 were used to probe N1 and C1, respectively. Plasmin-mediated α-cleavage showed a dose-dependent response (Figure 1 and Supplementary Figure 1).

### Plasmin generates C1 fragments in cultured cells

**FIGURE 2:**
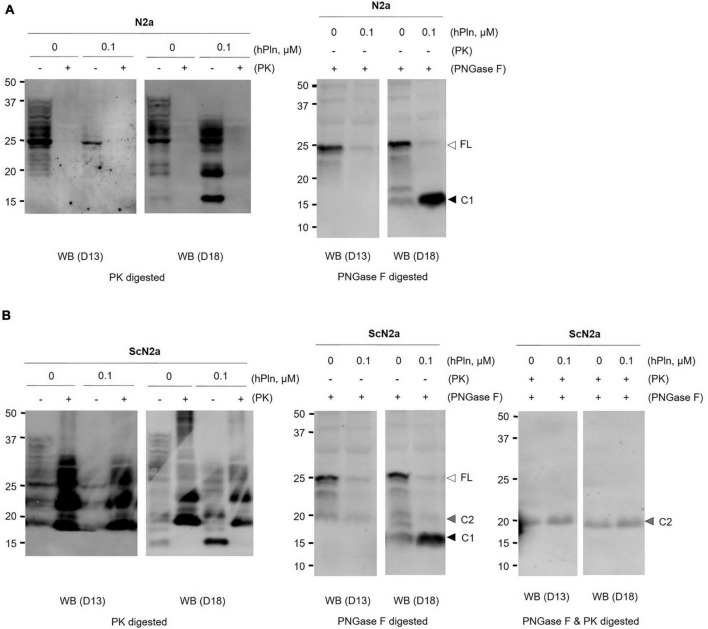
Plasmin cleaves PrP*^C^* in cultured cells. **(A)** N2a cells were grown with supplemented hPln and analyzed the level of C1. The increased signals in the D18 blot were sensitive to PK digestion. PNGase F treatment revealed the unglycosylated C1 fragment probed with D18. **(B)** Persistently scrapie-infected ScN2a cells were grown with supplemented hPln and analyzed the level of C1. The level of C1 was increased by hPln. The level of C2 was not affected by hPln. white arrow, full-length (FL) PrP*^C^*; black arrow, C1 fragment; gray arrow, C2 fragment.

To demonstrate the specific proteolytic activity of plasmin fragmenting PrP*^C^* in live cells, N2a and ScN2a cells were incubated with 0.1 μM of hPln. As shown in plasmin *in vitro* cleavage assays using rPrP, C1 formation was enhanced in the presence of hPln in both cells (Figure 2 and Supplementary Figure 2). N2a cells, a neuronal cell line with no prion infection, expressed variably glycosylated, GPI-anchored PrP*^C^* of full length and C1. hPln cleaved PrP*^C^*, generating variably glycosylated C1, which was recognized by D18 antibody (Figure 2A). Both PrP*^C^* of full length and C1 were sensitive to PK digestion. Increased formation of C1 by hPln was more obvious when glycans were removed from variably glycosylated PrP*^C^*. ScN2a cells persistently infected with prions produced PrP*^C^* and PrP*^Sc^* of full length and two endoproteolytic fragments C1 and C2. In ScN2a cells, hPln facilitated formation of endoproteolytic fragments of PrP*^C^*, which was detected by D18 antibody similarly to the events occurred in N2a cells. However, this did not cause a significant decrease of the level of PK-resistant PrP*^Sc^* (PrP27-30), which was recognized by both D13 and D18 antibodies (Figure 2B). When deglycosylated, increased formation of C1 by hPln was clearly demonstrated in ScN2a cells, too. When deglycosylated PrP and its fragments were hydrolyzed with PK, C2, equivalent to disease-specific PrP27-30, was recognized by both D13 and D18, and its level was unaltered by hPln. These results showed that plasmin involves in generation of C1, but not C2.

### Plasmin negates scrapie prion protein formation in scrapie-infected N2a cells

**FIGURE 3:**
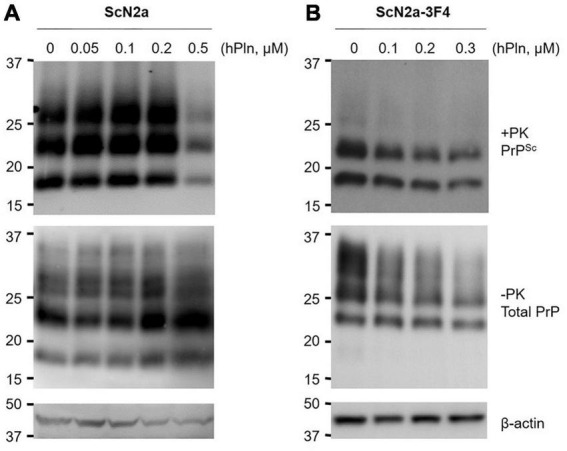
Plasmin reduces PrP*^Sc^* formation in cultured cells. ScN2a cells were grown in the presence of increasing concentrations of hPln. **(A)** The levels of total endogenous PrP (−PK) and PrP*^Sc^* (+ PK) accumulation in ScN2a cells cultured under the 10% FBS condition were evaluated by western blotting using anti-PrP antibody D13. **(B)** *De novo* formation of PrP*^Sc^* during hPln treatment was determined in ScN2a cells transiently transfected for PrP*^C^*-3F4 expression (ScN2a-3F4), which were cultured under the 2% FBS condition. PrP-3F4 (-PK) and PrP*^Sc^*-3F4 (+ PK) were specifically detected in western blots probed with anti-PrP antibody 3F4. β-actin was used as a reference protein to ensure equal amounts of each cell lysate were analyzed.

To determine whether plasmin-induced C1 generation inhibits PrP*^Sc^* formation under biological conditions, persistently prion-infected ScN2a cells were incubated with titrated concentrations of hPln for 6 d. PrP27-30 accumulation was significantly reduced by incubation with 0.5 μM hPln, although it appeared to be gradually increased by incubation with 0.05 – 0.2 μM hPln (Figure 3A and Supplementary Figure 3A). Incubation of ScN2a cells with hPln did not induce cell death within the concentrations employed in this experiment (data not shown). Total PrP and β-actin expression were stable, too, in all samples. To further understand how plasmin hinders PrP*^Sc^* accumulation, a similar experiment was conducted with cells transiently transfected to express 3F4-tagged PrP*^C^* [47] so that the function of plasmin on nascent PrP*^Sc^* formation could be determined. Following a period of 3 d, incubation with hPln demonstrated a dose-dependent decrease in newly generated 3F4-tagged PrP*^Sc^* and PrP*^C^* (Figure 3B and Supplementary Figure 3B).

### Scrapie prion protein generation in protein misfolding cyclic amplification is inhibited specifically by plasmin

**FIGURE 4:**
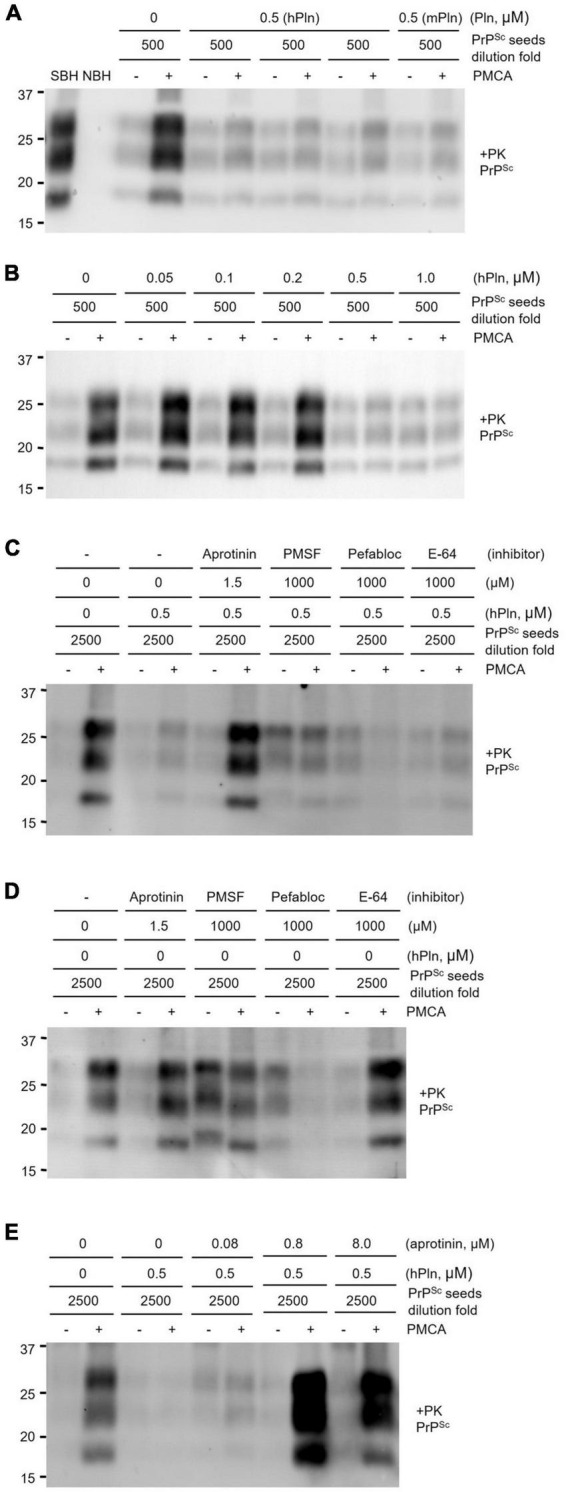
PMCA inhibited by supplementation with plasmin. Sick brain homogenate (SBH) were diluted 500 or 2500-fold in uninfected normal brain homogenate (NBH) and used as PrP*^Sc^* seeds for PMCA. PK digested pre- and post-PMCA samples are denoted by (−) and (+), respectively. PMCA products were evaluated by western blot analysis of PrP*^Sc^* generated. **(A)** PMCA supplemented with 0.5 μM plasmin of either human (hPln) or mouse (mPln) source was compared to identical reactions without supplementation. **(B)** PMCA conducted with increasing concentrations of hPln (ranging from 0 to 1 μM) was evaluated. **(C)** hPln-supplemented PMCA was performed in the presence or absence of protease inhibitors at an optimal working concentration for each. **(D)** PMCA with no hPln supplementation was performed in the presence or absence of protease inhibitors at an optimal working concentration for each. **(E)** hPln-supplemented PMCA was performed with increasing concentrations from 0 to 8 μM aprotinin.

An *in vitro* prion amplification technique was used to recapitulate the effects of plasmin in hPln-supplemented cell culture. PMCA was performed by diluting prion-infected brain homogenate 500-fold in that of a healthy animal. This dilution was chosen to evaluate plasmin because it consistently allowed robust PrP*^Sc^* production in PMCA, while a minute amount of PrP*^Sc^* could still be detected in the pre-amplification sample from the original seed. Under these conditions, the addition of 0.5 μM purified hPln and mPln successfully inhibited PrP*^Sc^* generation with no obvious preference for the sources (Figure 4A and Supplementary Figure 4A). However, PrP*^Sc^* propagation was not completely abolished and never reduced below levels of the original PrP*^Sc^* seed, as shown by a comparison of pre- and post-PMCA samples that were supplemented with plasmin. When similar PMCA was performed with titrated concentrations of hPln ranging from 0 to 1 μM, the amount of PrP*^Sc^* amplified was decreased in a dose-responsive manner (Figure 4B and Supplementary Figure 4B).

The PrP*^Sc^* product of hPln-supplemented PMCA performed in the presence of protease inhibitors, such as aprotinin, a specific serine protease inhibitor; PMSF, a broad-spectrum serine and cysteine protease inhibitor; Pefabloc Sc, a broad-spectrum serine protease inhibitor; and E-64, a selective cysteine protease inhibitor, was compared to determine whether the inhibitive effect was a function of plasmin. These protease inhibitors exhibit different specificity for plasmin. The inhibition constant (Ki) of aprotinin for plasmin is 4 nM (Fritz and Wunderer, 1983), which demonstrates aprotinin is most potent and specific among other inhibitors listed above. In fact, aprotinin was able to restore amplification of PrP*^Sc^* in hPln-supplemented PMCA, but not others (Figure 4C and Supplementary Figure 4C). In the control PMCA performed in the presence of each protease inhibitor but without hPln supplementation, aprotinin and E-64 did not affect PrP*^Sc^* generation by themselves, while Pefabloc SC failed PMCA (Figure 4D and Supplementary Figure 4D). Interestingly, PMSF appeared not to affect PMCA but to interfered with PK digestion of pre- and post-PMCA samples. Thus, plasmin-mediated inhibition of PrP*^Sc^* generation in hPln-supplemented PMCA was facilitated specifically by plasmin activity, which was suppressed by aprotinin. When hPln-supplemented PMCA was performed with titrated concentrations of aprotinin ranging from 0 to 8 μM, the amount of PrP*^Sc^* amplified was recovered in an aprotinin concentration-dependent fashion (Figure 4E and Supplementary Figure 4E).

### Inhibition of scrapie prion protein generation in protein misfolding cyclic amplification is attributed to plasmin-mediated endoproteolysis of cellular prion protein generating C1 fragments

**FIGURE 5:**
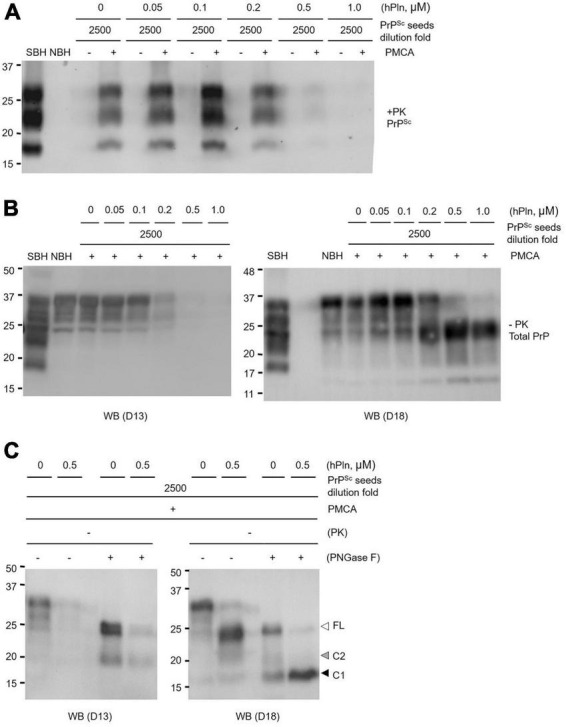
Plasmin inhibits PrP conversion in PMCA by promoting α-cleavage. Sick brain homogenate (SBH) were diluted 2500-fold in uninfected normal brain homogenate (NBH) and used as PrP*^Sc^* seeds for PMCA. PMCA products were evaluated by western blot analysis. **(A)** PMCA was conducted with increasing concentrations of hPln ranging from 0 to 1 μM and the level of PrP*^Sc^* was evaluated by western blot analysis with PK digestion. Pre- and post-PMCA samples are denoted by (-) and (+), respectively. **(B)** Post-PMCA (+) samples analyzed in Panel **(A)** were not subjected to PK digestion and further analyzed by western blotting with D13 or D18, recognizing full-length and fragments of PrP in different glycosylation states. **(C)** Post-PMCA (+) samples with no PK digestion from Panel **(B)** were deglycosylated by PNGase F and analyzed by western blot with D13 or D18. white arrow, full-length (FL) PrP; black arrow, C1 fragment; gray arrow, C2 fragment.

To eliminate detectable levels of PrP*^Sc^* from the seed, PMCA supplemented with titrated concentrations of hPln was repeated under conditions in which prion-infected brain homogenate was diluted 2500-fold in brain material from a healthy animal. Addition of 0.05 and 1 μM hPln caused a concentration-dependent decrease in the level of PrP*^Sc^* produced, where 0.5 and 1 μM hPln effectively prevented the appearance of newly formed PrP*^Sc^* (Figure 5A and Supplementary Figure 5A). To investigate plasmin-mediated internal cleavage of PrP, the hPln-supplemented PMCA samples without PK digestion were subjected to western blot analysis probed with D13 or D18. In the D13 blot, detection of PrP gradually diminished as the hPln concentration increased, presumably due to disappearance of D13 epitope by plasmin-mediated cleavage of full length PrP*^C^* and C2, which was not shown almost invisible in the blot because of its relatively low abundance. On the other hand, in the D18 blot, a band shift is observed as the hPln concentration increases, because hPln cleaved the full-length PrP*^C^*, resulting in an increase of C1 (Figure 5B and Supplementary Figure 5B). The results of the PNGase F treatment further confirmed that plasmin-mediated cleavage in PMCA altered C1 and C2 generation. PNGase F deglycosylation of the hPln-supplemented PMCA samples without PK digestion showed that hPln induced the production of C1 fragments (Figure 5C; compare lanes 7 and 8) and simultaneously reduced C2 production (Figure 5C and Supplementary Figure 5C; compare lanes 3 and 7 to 4 and 8, respectively).

### Supplemented plasmin reduces prion infectivity of protein misfolding cyclic amplification product

In addition to its effect to PrP*^Sc^* generation, the role of plasmin in controlling prion infectivity was assessed. Three groups of eight wild type mice received intracranial inoculation of the hPln-supplemented PMCA product, the non-supplemented PMCA product, or the PrP*^Sc^* seed used in PMCA reactions. All PMCA materials were generated by diluting prion-infected brain homogenate 16,000-fold in healthy brain material (Supplementary Figure 6). Infectivity of this material was estimated by comparing the mean incubation period from each group. According to statistical analysis, injection of material obtained from PMCA conducted in the presence of hPln resulted in an incubation time of 193 ± 4 d (mean incubation time ± standard error). This incubation period was significantly longer than that of the non-supplemented PMCA product (177 ± 2 d; *P* < 0.001), but statistically equivalent to that of the PrP*^Sc^* seed (198 ± 8 d; *P* > 0.05) (Table 1).

**TABLE 1:** Bioassay of PMCA materials with or without supplementation with plasmin.

Sample	No. Mice (Prion-sick/Total)	Avg. Incubation Time ± SEM (days)	*P*-value (vs. PMCA)	*P*-value (vs. PrP*^Sc^* Seeds)
PrP*^Sc^* Seeds	7/8	198 ± 8	0.01	
PMCA	8/8	177 ± 2		
PMCA + hPln	8/8	193 ± 4	< 0.001	NS (> 0.05)

Western blot analysis of PK-resistant PrP*^Sc^* in the mouse brains of all three groups showed that PrP*^Sc^* accumulated in the end-stage animals ill with prion disease (Supplementary Figure 7). The neuropathology of the mouse brains of each group also supported that the prion-ill mice were due to prion disease with vacuolation and gliosis in their brains which are typical signs of prion disease (Supplementary Figures 8, 9).

## Discussion

### Rationale

All members of the PrP superfamily (PrP, Shadoo, and Doppel) have been shown to occupy similar membrane environments and undergo congruent endoproteolytic events (Mays et al., 2014a). Transition in metabolic processing for PrP was originally described for CJD patients nearly three decades ago and has since been documented as being altered in other models of prion disease (Chen et al., 1995; Jimenez-Huete et al., 1998; Yadavalli et al., 2004; Ayers et al., 2011). In the healthy state, a proportion of mature PrP*^C^* undergoes endoproteolysis at an α-cleavage site that presumably occurs N-terminal to the hydrophobic domain generating a GPI-anchored C-terminal fragment (C1) and subsequently releasing an N-terminal fragment (N1). However, during prion disease, PrP more favorably undergoes alternative β-cleavage C-terminal to the octarepeat that yields a larger C-terminal fragment (C2) and its corresponding N-terminal fragment (Chen et al., 1995; Jimenez-Huete et al., 1998). Interestingly, C2 is equivalent in size to PrP27-30, which is the protease-resistant core of PrP*^Sc^*. Although the exact cleavage site has not yet been elucidated, γ-cleavage appears to be associated with pathophysiological conditions, as increased C3 is found in CJD brain samples (Lewis et al., 2016). Therefore, the initial processing of PrP*^C^* at the α-cleavage site to produce the C1 has long been considered as a potential prophylactic for prion diseases by preventing the production of infectious and/or toxic forms of PrP.

### Plasmin inhibits prions by specifically cleaving cellular prion protein

Successful generation of the PrP C1 in various model systems has been shown for ADAM8 (Liang et al., 2012), ADAM10 (Vincent et al., 2001; Cisse et al., 2005), ADAM17 (Vincent et al., 2001; Laffont-Proust et al., 2005), calpain (Hachiya et al., 2011), and plasmin (Kornblatt et al., 2003; Praus et al., 2003; Xanthopoulos et al., 2005). However, the *bona fide* protease responsible is currently a matter of debate because recent evidence questions the prominence in which the leading candidates, ADAM10 and ADAM17, play in the α-cleavage of PrP. In summary, there has been large discrepancy in the amount of C1 obtained from cultured cells over-expressing or depleted of ADAM10 or ADAM17 (Vincent et al., 2000, 2001; Taylor et al., 2009; Béland et al., 2012). Moreover, neuronal over-expression or knockdown of ADAM10 *in vivo* failed to influence α-cleavage (Endres et al., 2009; Altmeppen et al., 2011). In contrast, a convincing case was reported describing ADAM8 as the primary protease generating C1 in skeletal muscle *in vitro* and *in vivo*, but this likely cannot be recapitulated in the central nervous system due to negligible expression levels (Su et al., 2004; Liang et al., 2012). Lastly, a role for calpain contradicts the hypothesis that C1 and C2 are cleaved by separate pathways since it has demonstrated the capacity to perform α- and β-cleavage *in vitro* (Yadavalli et al., 2004).

Here, we focused on the unexplored function of plasmin in PrP endoproteolysis during PrP*^Sc^* formation and prion propagation. Although primarily found in the liver, plasminogen is expressed by neuronal populations in the brain and often localizes to the lipid rafts of the plasma membrane (Ledesma et al., 2003; Kim et al., 2009). Therefore, this observation indirectly places plasmin at the site for PrP*^Sc^* replication (Vey et al., 1996) because plasminogen readily converts into plasmin. To better understand the role of plasmin, we used an *in vitro* plasmin cleavage assay to recognize the nature of plasmin responsible for the C1 cleavage. We tested the specific proteolytic activity of plasmin by using rhPrP(23-231) in the presence of 10% FBS and PrP*^C^* expressing-neuronal cell lines with or without chronic prion infection. In the present study, we confirmed that plasmin has the fundamental ability to internally cleave PrP at the α-cleavage site in agreement with previous reports (Figures 1, 2). Inclusion of FBS in the rhPrP(23-231) cleavage reaction, which was not the case for previous reports, was carried out in this study to investigate the cleavage under a condition that is similar to the physiological environment. Because serum includes a number of regulators that control plasmin activity, the result of hPln-mediated rhPrP(23-231) cleavage in the presence of serum strongly suggests that the generation of rhPrP(23-231) fragments by hPln is not artifacts obtained under the simple *in vitro* condition. This is further expanded in the cultured cells. Plasmin increased formation of the C1, while analysis of deglycosylated PrP showed that the level of C2 was not changed by plasmin treatment in ScN2a cells.

To address that plasmin induction of C1 generation would inhibit PrP*^Sc^* formation under biological conditions, ScN2a and PrP-3F4 expressing ScN2a cells were exposed to plasmin. In ScN2a cells, PrP*^Sc^* accumulation was significantly, but suddenly, reduced by incubation with 0.5 μM hPln, while it was gradually increased by incubation with low concentrations of hPln (Figure 3A). This indicates that there is a threshold of hPln concentration to facilitate inhibition of PrP*^Sc^* formation under the experimental conditions used for this study. Because ScN2a cells were cultured in the presence of 10% serum that includes the natural plasmin inhibitor such as α2-antiplasmin estimated to be 0.2 μM (Cederholm-Williams, 1981), the supplemented hPln at the low concentrations could remain inactive to cleave PrP*^C^*. Interestingly, this inactive plasmin harboring Kringle domains could be virtually identical to plasminogen that enhances PrP*^Sc^* formation as we reported previously (Ryou et al., 2003). Therefore, it is likely that PrP*^Sc^* accumulation is stimulated until, but inhibited after, the concentration of hPln exceeds the level of natural plasmin inhibitors provided in serum. The inhibition of PrP*^Sc^* accumulation found in ScN2a cells was likely due to the inhibition of *de novo* PrP*^Sc^* formation as shown in ScN2a cells transiently transfected for PrP-3F4 expression (Figure 3B). From these results, we suggested that plasmin would inhibit the conversion of PrP*^C^* to PrP*^Sc^* by generating C1 lacking the neurotoxic and amyloidogenic PrP(106-126) domains without a direct effect on C2 alteration. Other studies showed that the reduction of the PrP*^C^* level resulted in decreased prion replication during the transition from presymptomatic to symptomatic prion disease (Mays et al., 2014b,^2015^). Interestingly, the level of total PrP in ScN2a expressing PrP-3F4 was decreased as the concertation of supplemented hPln increased, implicating the reduction of the PrP*^C^* level (Figure 3B). Thus, plasmin-mediated cleavage of PrP*^C^* could potentially contribute to the diminishing residual full-length PrP*^C^* levels in the presymptomatic to symptomatic period transition.

PMCA provided a simplified system to study PrP*^Sc^* formation in the presence of physiologically relevant plasmin levels (Cederholm-Williams, 1981; Whitelaw et al., 1995), while avoiding the complex pathways necessary to activate plasminogen to plasmin (Castellino and Ploplis, 2005) and intrinsic health problems associated with the plasminogen knockout mouse model (Ploplis et al., 1995; Bugge et al., 1995) that has confounded the interpretation of prion bioassays in the past. The inhibition of PrP*^Sc^* generation by plasmin was confirmed using PMCA in this study (Figures 4A,B). The plasmin function could only be abolished with the addition of aprotinin, a serine protease inhibitor specific and potent for plasmin (Figures 4C–E), suggesting an apparent role of plasmin as a negative regulator in PrP*^Sc^* generation. The role of plasmin to inhibit PrP*^Sc^* generation was extended to its role in inhibiting prion infectivity from the bioassay using PMCA materials produced in the presence or absence of hPln (Table 1). In this study, the material obtained from PMCA resulted in the abbreviated incubation time over 21 d compared to the PrP*^Sc^* seeds, the material without amplification, suggesting an increase of prion titer by PMCA. The plasmin supplementation in PMCA counteracted the effect of amplification and, like the PrP*^Sc^* seeds, the incubation time of this material prolonged 16 d compared to the material obtained from PMCA without hPln supplementation. This delay of incubation time represents the abolition of PMCA prion titer by plasmin to the level of the PrP*^Sc^* seeds because the incubation time between the PrP*^Sc^* seeds and hPln-supplemented PMCA groups was not different with statistical significance. Thus, plasmin supplementation in PMCA efficiently inhibited infective prion propagation by producing an endoproteolytic fragment of PrP*^C^* (Figure 5), which can serve as improper substrates for conversion, via α-cleavage. Because the substrate of normal brain homogenates used for PMCA was prepared in PBS with the protease inhibitor cocktail that includes the agents inhibiting plasmin, the α-cleavage of PrP*^C^* by plasmin and the subsequent PrP*^Sc^* formation may not be robust under the experimental conditions in this study.

The mechanistic details underlying the endoproteolysis of PrP*^C^* by plasmin remain to be explored. PrP*^C^* is known to interact with plasminogen and tissue-type plasminogen activator (tPA) (Fischer et al., 2000; Maissen et al., 2001; Ellis et al., 2002; Shaked et al., 2002; Kornblatt et al., 2003, 2004; Praus et al., 2003; Ryou et al., 2003; Epple et al., 2004a,b; Cuccioloni et al., 2005; Hatcher et al., 2009; Bougard et al., 2016). Because plasmin is activated from plasminogen by tPA, this event can occur in a complex with PrP*^C^*, which enables plasmin readily cleaves PrP*^C^* at the α-site. The substrate specificity of PrP*^C^* for plasmin catalysis has not been documented. However, although not perfect, the α-site of PrP*^C^* resembles the arrangement of amino acid residues (Xaa-Tyr/Phe-Lys/Arg-Xaa, where Xaa can be any amino acid) preferred by plasmin (Harris et al., 2000). Like many serine proteases, plasmin cleaves the peptide bond between Lys/Arg-Xaa, leaving a C-terminal lysine, with a preference of an aromatic amino acid reside at the N-terminal of Lys/Arg. The α-site of human and mouse PrP*^C^* shows Asn-Met/Leu-Lys-His. Although aliphatic Met/Leu are not aromatic, they share the hydrophobic characteristics with Tyr/Phe and could fill the space of a specificity pocket near the catalytic triad of plasmin similar to Tyr or Phe does. Thus, PrP*^C^* may not be the best substrate for plasmin, while it is sufficient enough to be cleaved by plasmin, which contributes to the prevention of PrP*^Sc^* formation as demonstrated in the current study.

### Implications for plasmin in neurodegenerative diseases

Defining the role of plasmin in the endoproteolysis of PrP also may have implications for other neurodegenerative diseases on multiple levels. First, the suggested involvement of multiple pathways for the metabolic processing of PrP parallels the situation in the healthy, non-amyloidogenic α-processing versus the disease-associated amyloidogenic β-processing of the β–amyloid precursor protein (βAPP) (Selkoe, 1999; Venugopal et al., 2008). Secondly, enzymatic assays demonstrated that plasmin intervenes by scission of APP at the α-cleavage site between Lys687 and Leu688 (Selkoe, 1999) as well as by degrading Aβ40 and Aβ42 via cleavage between Arg5 and His6 or at multiple other sites (Van Nostrand and Porter, 1999; Tucker et al., 2000a,b; Exley and Korchazhkina, 2001). Concomitantly, induced activation of plasminogen into plasmin has been indicated to reduce Aβ levels *in vivo* (Melchor et al., 2003; Tucker et al., 2004; Jacobsen et al., 2008; Liu et al., 2011). Thirdly, PrP*^C^* contains a binding site for oligomeric Aβ located between the C1 and C2 sites, which has been shown to mediate toxicity (Lauren et al., 2009; Westaway and Jhamandas, 2012). Therefore, plasmin α-cleavage of PrP generates a GPI-anchored C1 in which oligomeric Aβ could not dock, thus preventing its toxic effect during Alzheimer’s disease. It is also suggested that the proteolytic activity of plasmin is associated with Parkinson’s disease. Plasmin is capable of cleaving and degrading α-synuclein in both its monomeric and aggregated forms, inhibiting the translocation of extracellular α- synuclein into the neighboring cells, and reducing the neuroinflammatory response of microglia and astrocytes. This prevents α-synuclein from aggregating and forming toxic Lewy bodies, resulting in reduced neuronal cell death (Kim et al., 2012; Park and Kim, 2013). Although the role of plasmin/plasminogen in the pathogenesis of amyotrophic lateral sclerosis remains to be elucidated, several studies have reported that plasminogen/plasmin is associated with the pathogenesis of amyotrophic lateral sclerosis (Demestre et al., 2006; Glas et al., 2007).

## Summary and conclusion

In summary, we demonstrated an inhibitory role for plasmin in PrP*^Sc^* formation using PMCA and observed a parallel *de novo* reduction in the accumulation of PrP*^Sc^* in ScN2a cells incubated with plasmin. Although it remains to be determined whether plasmin inhibits PrP*^Sc^* propagation during natural prion infectivity *in vivo*, plasmin inhibits propagation of infective prion replication during PMCA as shown in bioassays of PMCA products. While the ability to generate the PrP C1 fragment is shared by several proteases, our data presents plasmin as the first discovered to play a functional role during prion formation by cleaving PrP*^C^*, but not PrP*^Sc^*. Moreover, the present study complements our recent discovery that plasminogen assists in PrP*^Sc^* propagation by introducing a novel regulatory role for the plasmin(ogen) system that could not be revealed in complex plasminogen knockout mouse models. Although the mechanistic details are uncertain, the “yin-yang” effect of the plasmin(ogen) system not only contributes to deciphering the intricate events involved in prion replication but presents new and attractive therapeutic targets to treat prion diseases as well as other neurodegenerative diseases.

## Data availability statement

The original contributions presented in this study are included in the article/Supplementary material, further inquiries can be directed to the corresponding authors.

## Ethics statement

The animal study was reviewed and approved by Institutional Animal Care and Use Committee, University of Kentucky.

## Author contributions

CM and TT performed the experiments. CM, TT, GT, H-EK, and CR analyzed the data. CM, H-EK, and CR wrote the manuscript. All authors read and approved the final manuscript.
