# Supplementary material for: Endoproteolysis of cellular prion protein by plasmin hinders propagation of prions
Source: Front Mol Neurosci. 2022 Sep 2;15:990136. doi: 10.3389/fnmol.2022.990136 (PMC9478470; doi:10.3389/fnmol.2022.990136)
Supplement: Supplementary file 1 [file Data_Sheet_1.docx]

**Supplementary information**

**Endoproteolysis of PrP^C^ by plasmin hinders propagation of prions**

Charles E. Mays^1^, Trang H. T. Trinh^2,3^, Glenn Telling^1,4,5^, Hae-Eun Kang^5,6*^, and Chongsuk Ryou^1,2,3,4*^

^1^Department of Microbiology, Immunology, and Molecular Genetics, University of Kentucky College of Medicine, Lexington, Kentucky, USA

^2^Department of Pharmacy, College of Pharmacy, Hanyang University, Ansan, Republic of Korea

^3^Institute of Pharmaceutical Science and Technology, Hanyang University, Ansan, Republic of Korea

^4^Sanders-Brown Center on Aging, University of Kentucky College of Medicine, Lexington, Kentucky, USA

^5^Department of Microbiology, Immunology, and Pathology, Colorado State University, Fort Collins, Colorado, USA

^6^Reference Laboratory for Chronic Wasting Disease (CWD), Foreign Animal Disease Division, Animal and Plant Quarantine Agency, Gimcheon, Republic of Korea

Running title: Inhibition of prion propagation by plasmin-induced PrP^C^ cleavage

***Correspondence:**

Chongsuk Ryou, cryou2@hanyang.ac.kr
Hae-Eun Kang, kanghe@korea.kr

**
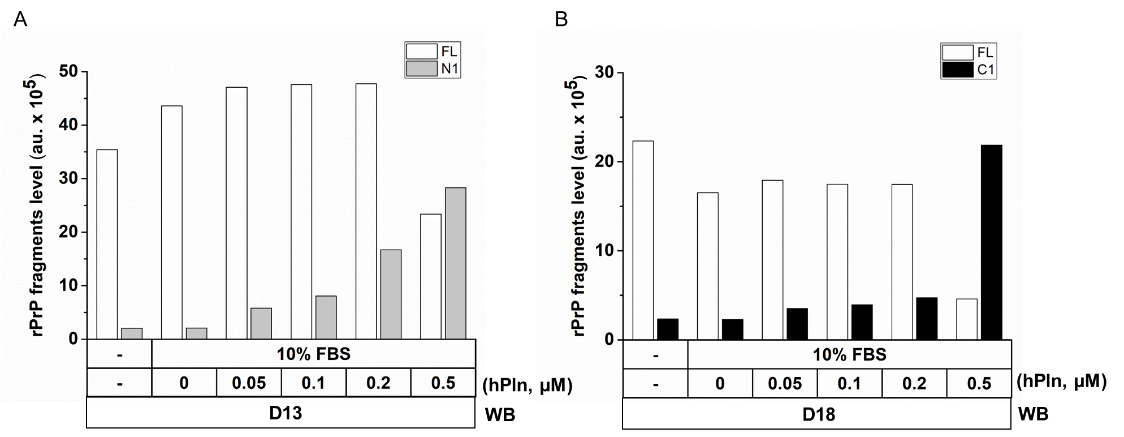
**

**Figure S1. Quantification** **of rhPrP (23-231) fragments generated by plasmin in the serum conditioned buffer.** Densitometry of PrP fragments shown in Figure 1. (A) Full length (FL, open bar) and N1 (light gray bar) fragments detected by western blot using D13 antibody, (B) Full length (FL, open bar) and C1 (filled bar) fragments detected by western blot using D18 antibody. au, arbitrary unit.

**
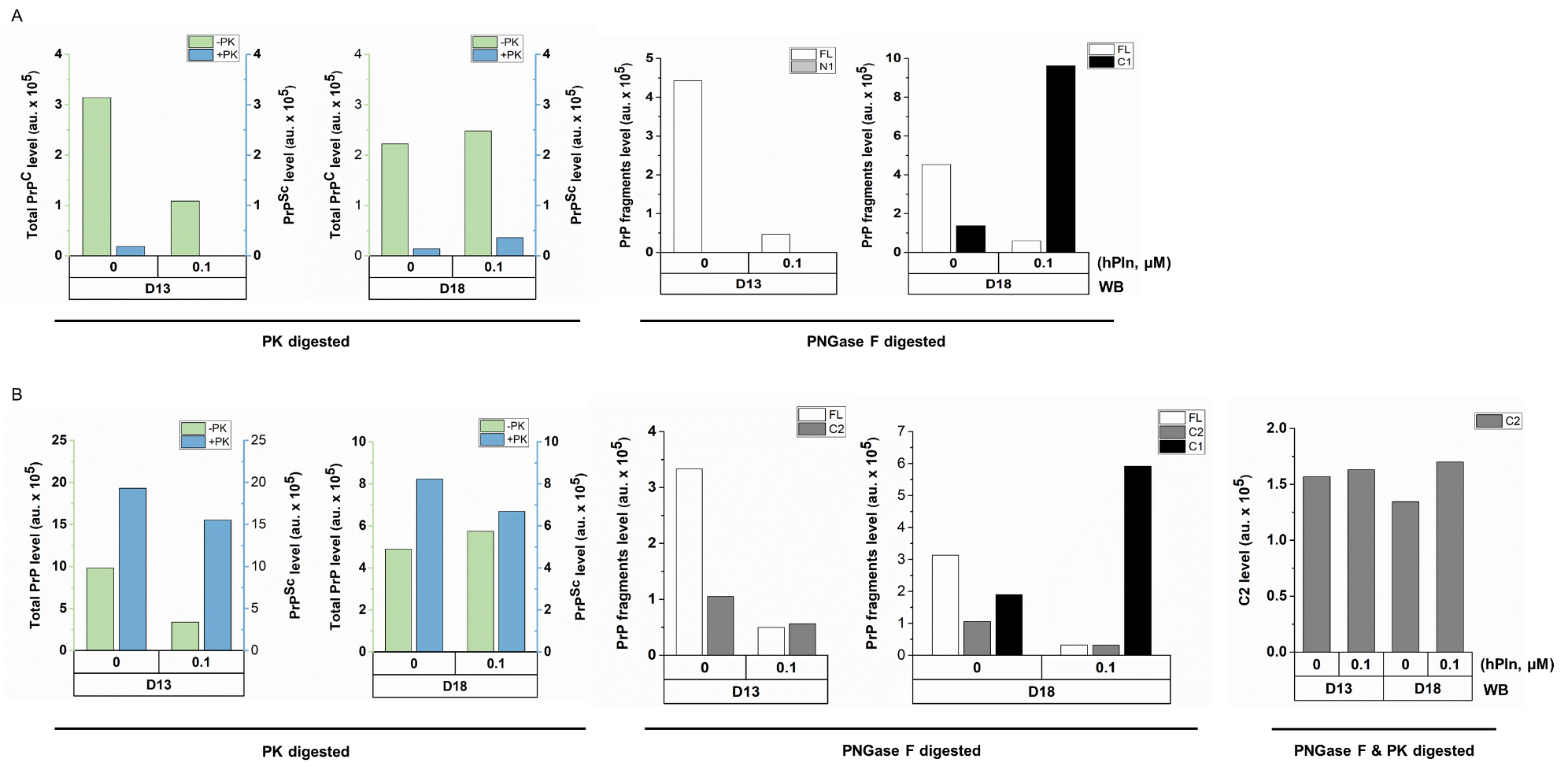
**

**Figure S2. Quantification of PrP fragments generated by plasmin in cell culture.** Densitometry of PrP fragments shown in Figure 2. (A) In N2a cells, total level of PrP^C^ (green bar, -PK), PK-resistant (blue bar. +PK), full length (FL, open bar), N1 (light gray bar), and C1 (filled bar) PrP species were compared. (B) In ScN2a cells, total PrP (green bar, -PK), PK-resistant PrP^Sc^ (blue bar. +PK), full length (FL, open bar), C2 (dark gray bar), and C1 (filled bar) PrP species were compared. Each colored bar is read with the y-axis with the identical color. au, arbitrary unit.

**
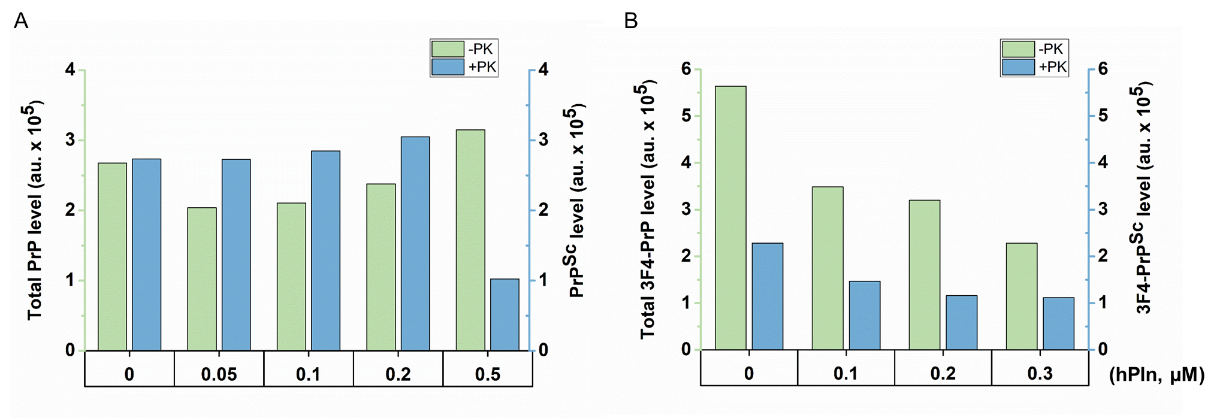
**

**Figure S3. Quantification of the PrP^Sc^ level in ScN2a cells cultured in the presence of plasmin.** Densitometry of total PrP and PK-resistant PrP^Sc^ shown in Figure 3.(A) Level of total PrP (green bar, -PK) and PrP^Sc^ (blue bar, +PK) of ScN2a cells cultured with various concentrations of human plasmin (hPln) were detected by western blotting using D13 antibody. (B) Level of newly synthesized PrP^C^-3F4 and PrP^Sc^ -3F4 of ScN2a clles overexpressing 3F4-tagged PrP^C^ cultured with various concentrations of hPln. 3F4-tagged PrP species were detected using anti-PrP antibody 3F4. Each colored bar is read with the y-axis with the identical color. au, arbitrary unit.

**
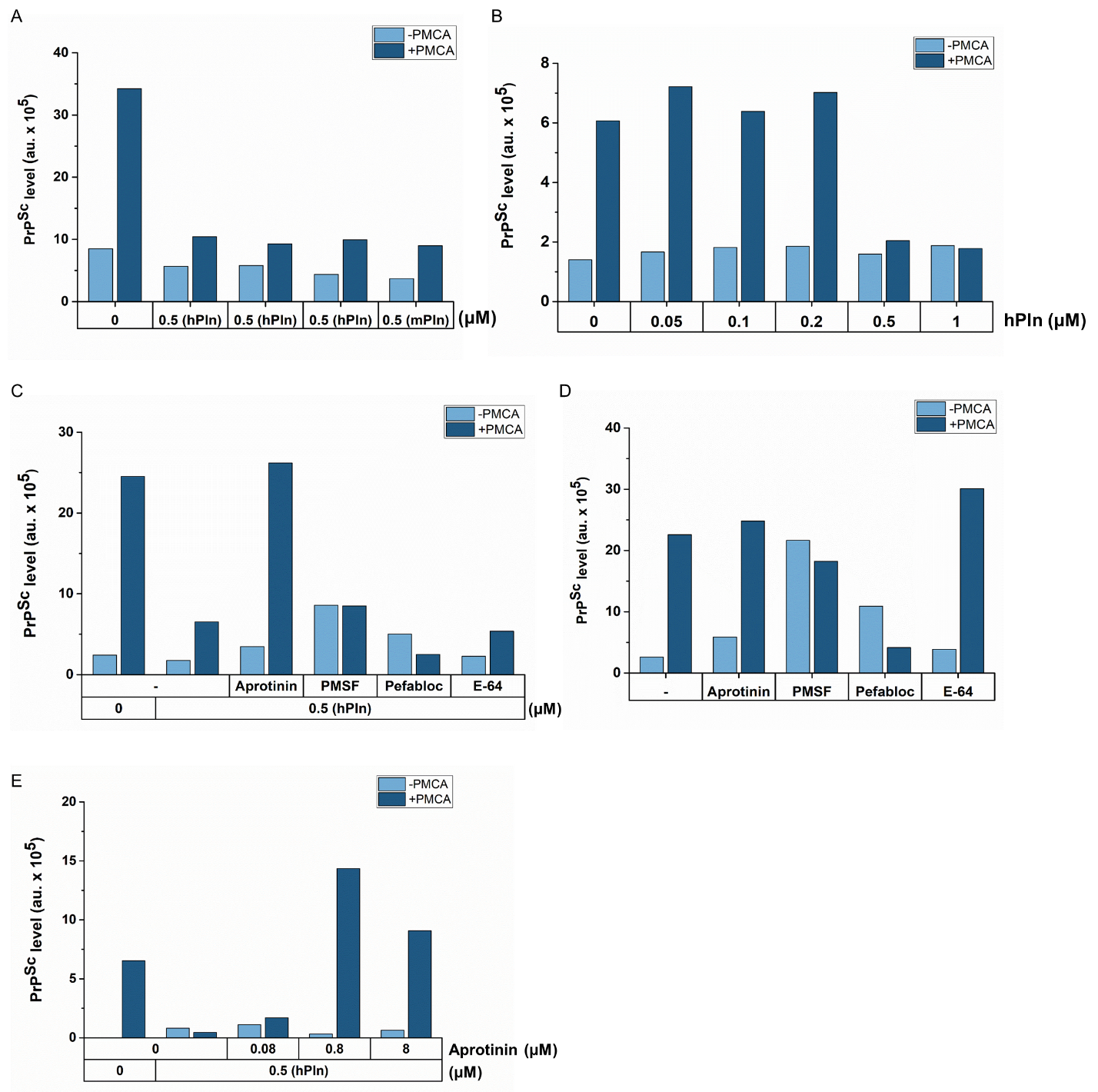
**

**Figure S4 Quantification of PK-resistant PrP^Sc^ level in PMCA supplemented with plasmin.** Densitometry of PK-resistant PrP^Sc^ shown in Figure 4. (A) Level of PrP^Sc^ in PMCA conducted in the presence of 0.5 μM human (hPln) or mouse (mPln) plasmin. (B) PrP^Sc^ in PMCA with increasing concentrations of hPln. (C) Level of PrP^Sc^ generated in PMCA supplemented with different protease inhibitors and 0.5 μM hPln. (D) Level of PrP^Sc^ in PMCA supplemented with different protease inhibitors but no hPln. (E). Level of PrP^Sc^ in hPln-supplemented PMCA performed with increasing concentrations of aprotinin. –PMCA, pre-PMCA sample (light blue bar); +PMCA, post-PMCA sample (dark blue). au, arbitrary unit.

**
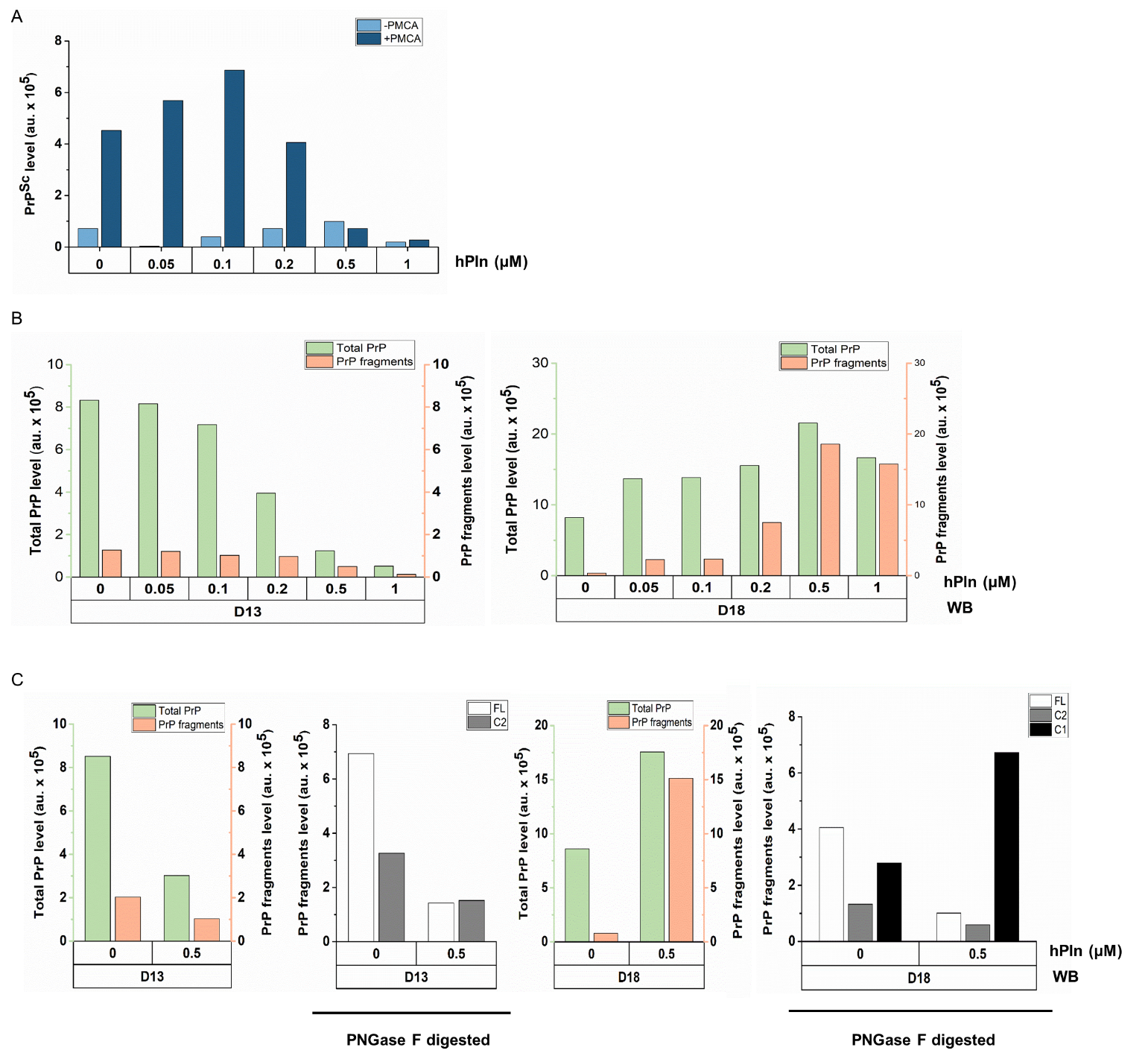
**

**Figure S5 Quantification of PrP fragments generated in plasmin-supplemented PMCA.** Densitometry of PrP fragments in PMCA shown in Figure 5. (A) Level of PrP^Sc^ in (-) PMCA (light blue bar) and (+) PMCA (dark blue) supplemented with increasing concentrations of hPln. (B) and (C, first and third panels) Total level of PrP (green bar) and PrP fragments (light orange bar) that contain D13-epitope (left panel), and D18-epitope (right panel) in hPln-supplemented PMCA products. Each colored bar is read with the y-axis with the identical color. (C, second and fourth panels) Level of deglycosylated form of full length (FL, open bar) PrP, C2 fragment (dark gray bar) and C1 fragment (filled bar) in hPln-supplemented PMCA products detected by D13 and D18 antibodies. au, arbitrary unit

**
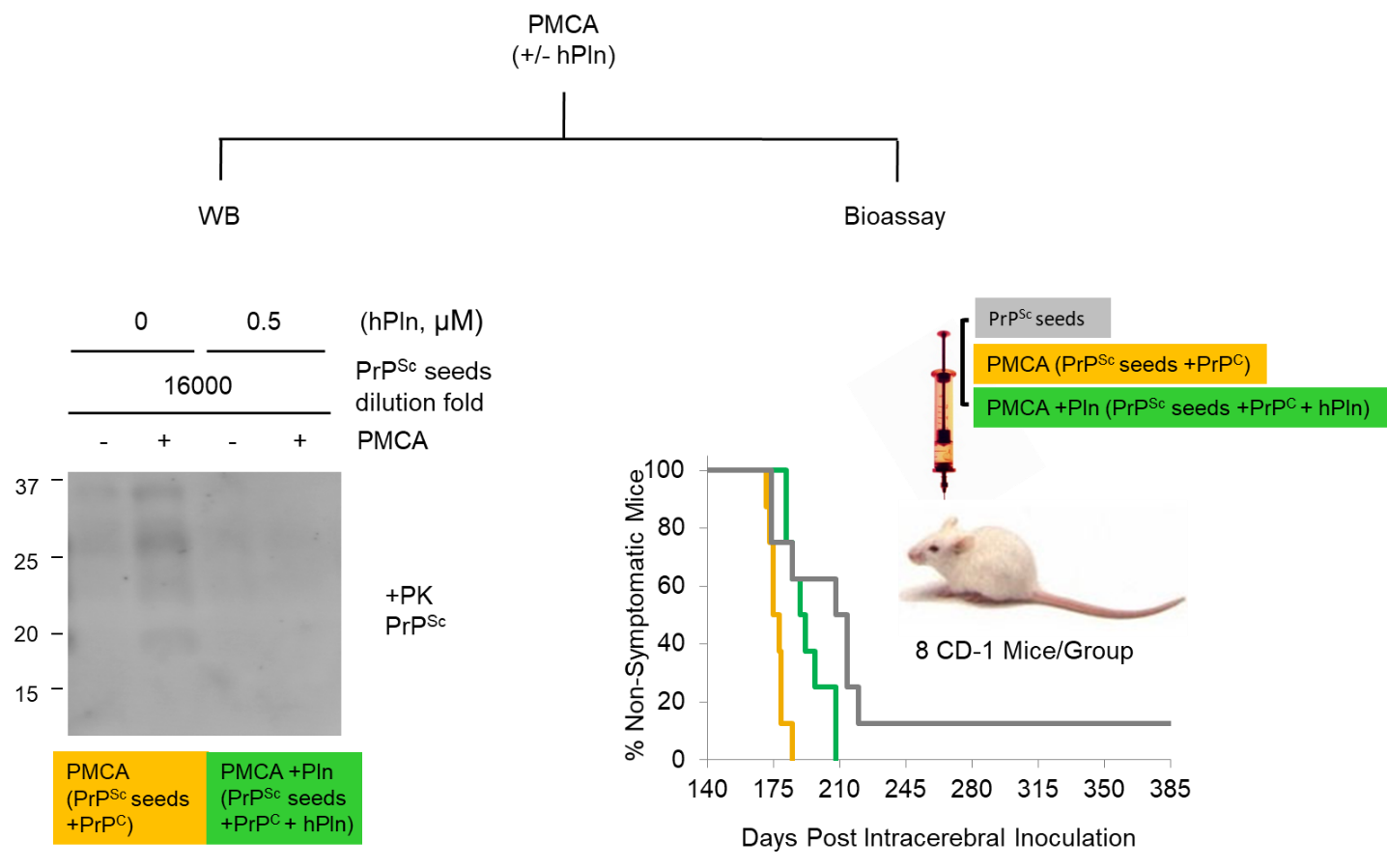
**

**Figure S6. Bioassay of PMCA material generated with plasmin**. PMCA was performed with RML-infected brain homogenate diluted 1:16000 in brain material from healthy CD-1 mice. hPln (0.5 µM)-supplemented PMCA material was analyzed by either western blotting or bioassay. In western blot, supplementation of hPln in PMCA interfered PK-resistant PrP^Sc^ generation in post-PMCA (+) samples to the level found in PrP^Sc^ seeds of pre-PMCA (-) sample, although the PrP^Sc^ level was near the detection limit due to highly diluted PrP^Sc^ seeding. In bioassay, each PMCA material (PMCA supplemented with or without hPln) was diluted 1:10 in PBS and 30 µl was intracerebrally injected into CD-1 mice (8 mice/group). Diluted and identical volume of RML-infected brain homogenate equivalent to the amount of PrP^Sc^ seeds used for PMCA was injected into mice by the same manner. The mice were allowed to reach the time at which multiple disease signs began to progress simultaneously. The disease onset within each group was plotted by Kaplan-Meier method (Refer to Table 1). A living animal with no clinical signs from PrP^Sc^ seed group was euthanized at 385 d post inoculation. Each group was color-coded to correlate the bioassay results and injected inocula, as well as the result of western blotting.


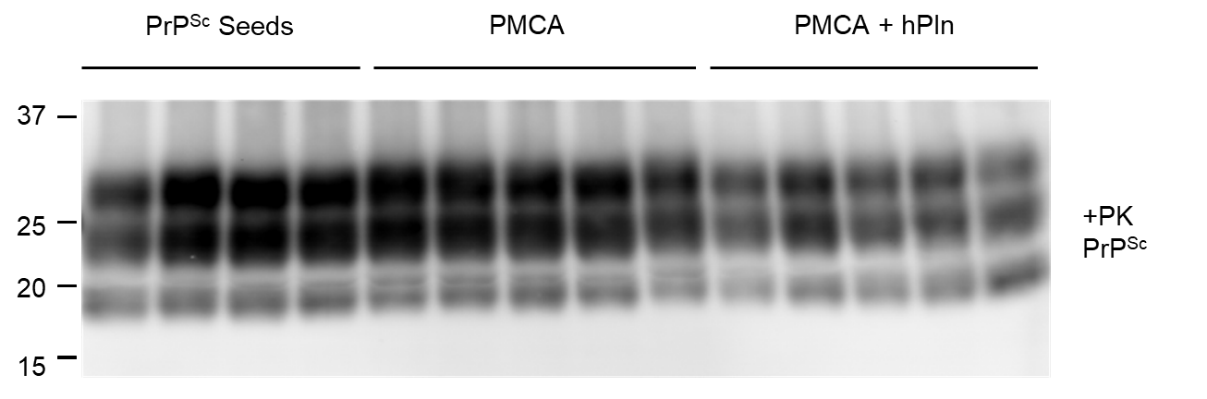


**Figure S7. Western blot of accumulated PrP^Sc^ in sick mouse brains collected from each group of bioassay**. Mice with clinical signs of prion disease in each group were euthanized and the presence of PK-resistant PrPSc in their brains was detected by western blotting using anti-PrP antibody D13. Although it appears that the PrP^Sc^ level fluctuate among samples and groups, all samples demonstrated the presence of a considerable level of PrP^Sc^ because all samples were collected from mice at the terminal stage of disease, although the days required for onset varied.

**
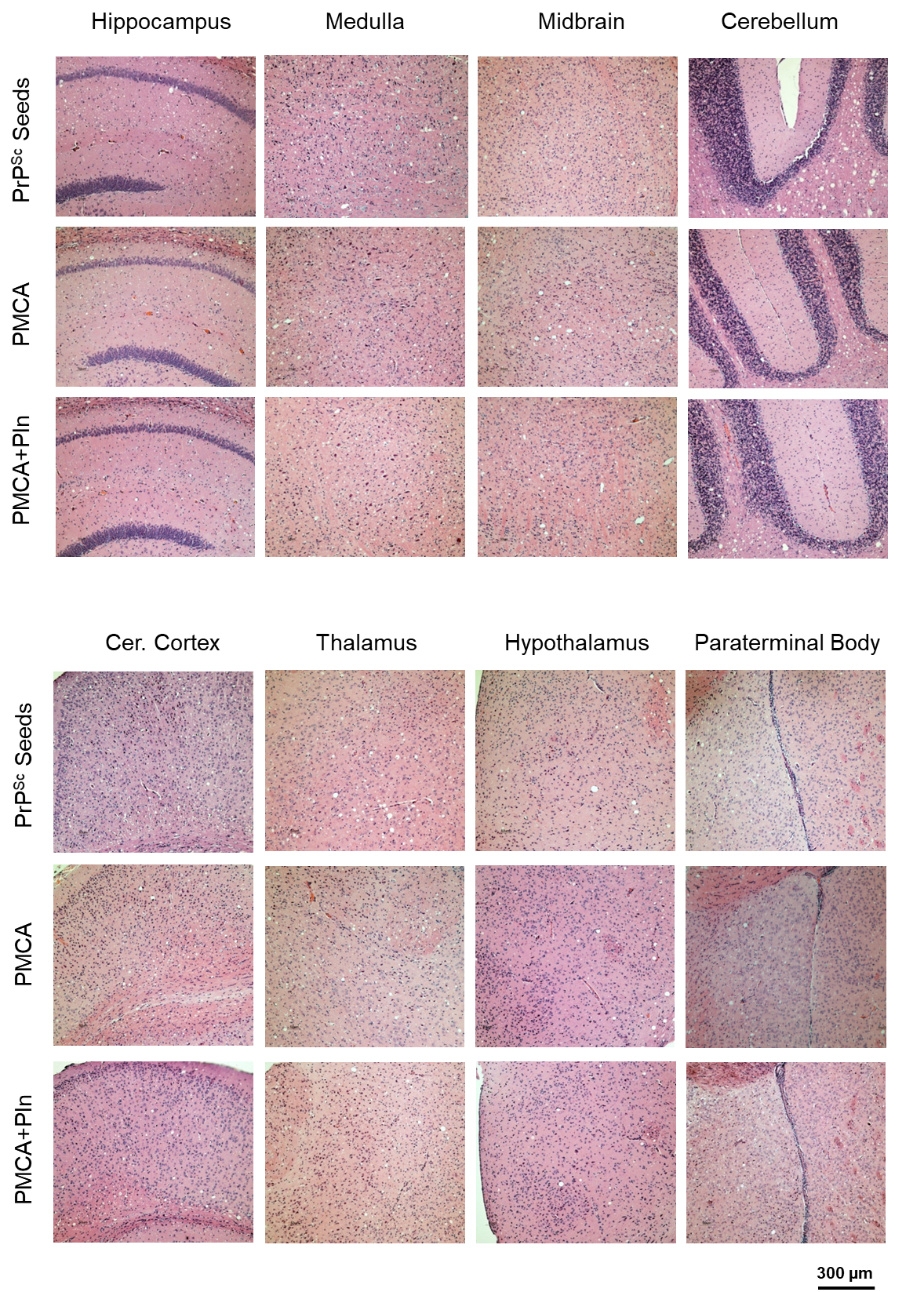
**

**Figure S8. Vacuolations in the sick mouse brains collected from each group of bioassay.** The coronal sections of mouse brains were H&E stained. Different regions of brain from each group showed the presence of a considerable number of vacuoles, indicating the onset of prion disease in mice. Scale bar: 300 μm.

**
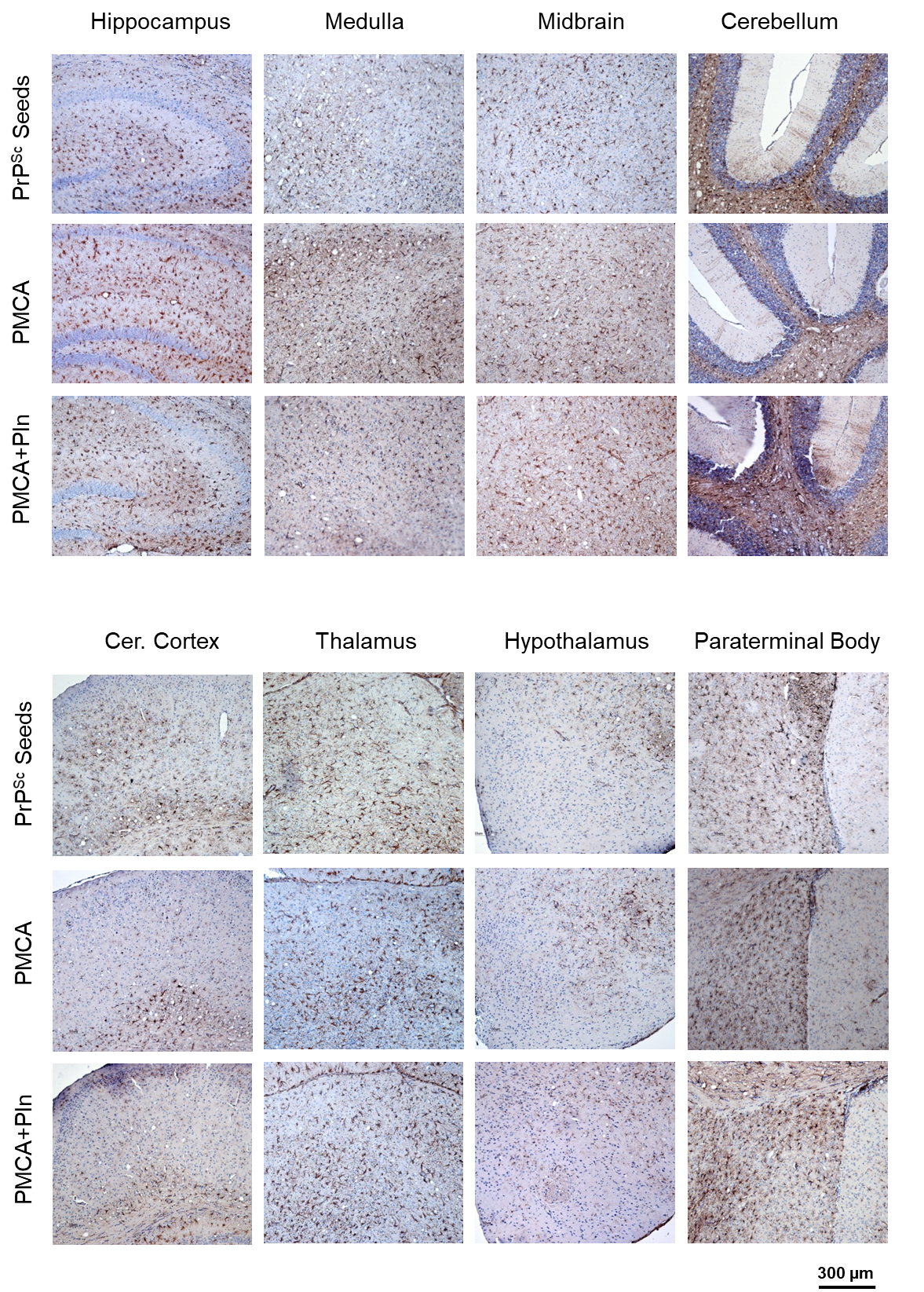
**

**Figure S9. Astrogliosis in the sick mouse brain collected from each group of bioassay.** GFAP was stained by immunohistochemistry in different regions of brain section of mice. Staining of a considerable level of GFAP expression indicates the progress of prion disease in mice. Scale bar: 300 μm.
